# Assessment of the Level of Knowledge and Attitude Towards Herpes Zoster and Its Vaccination Among Individuals at Risk in Saudi Arabia

**DOI:** 10.7759/cureus.53572

**Published:** 2024-02-04

**Authors:** Nasser Al Shanbari, Asayel Aldajani, Fatoon Almowallad, Wafa Sodagar, Hatim Almaghrabi, Nada S Almuntashiri, May Alshareef, Faisal M ALzubaidi, Mokhtar Shatla

**Affiliations:** 1 Department of Medicine and Surgery, College of Medicine, Umm Al-Qura University, Makkah, SAU; 2 Department of Community Medicine and Healthcare of Pilgrims, Umm Al-Qura University, Makkah, SAU

**Keywords:** public heath, varicella vaccine, varicella-zoster, chickenpox, herpes zoster

## Abstract

Background

Herpes zoster (HZ) is a viral infection resulting from the reactivation of the varicella-zoster virus. The vaccination was proven to prevent herpes zoster and its complications for individuals over the age of 50 since they are more susceptible to herpes zoster infection. Therefore, it is essential to understand and acknowledge the herpes zoster infection and vaccine. This study aims to assess the level of knowledge and attitude towards herpes zoster and its vaccination among individuals at risk in Saudi Arabia.

Methods

A cross-sectional study was conducted between February 2023 and June 2023 among the general population in Saudi Arabia, using a self-administered online questionnaire adapted from a previous study after translating it into Arabic.

Results

A total of 1883 participants were included in this study, almost equally distributed across the five regions of Saudi Arabia. Females represented 62.4% (n=1175), and 56% (n=1062) of participants reported a positive history of chickenpox, while 3.6% (n=67) reported a history of herpes zoster. Furthermore, 78.9% (n=1486) have heard of herpes zoster, while 68.8% (n=1296) have at least one of the herpes zoster risk factors. Only 7.8% (n=147) had high knowledge about HZ, and only 3.1% (n=58) had adequate knowledge regarding the HZ vaccine.

Conclusion

Our study findings show that the general population of Saudi Arabia has inadequate knowledge about herpes zoster and its vaccine. For that reason, awareness and education programs targeting individuals at high risk of herpes zoster are required to enhance awareness and knowledge about herpes zoster and to improve their attitudes toward the herpes zoster vaccination.

## Introduction

Herpes zoster (HZ) is a viral infection characterized by localized, painful vesicular rash limited to one or two adjacent dermatomes; it results from the reactivation of latent varicella-zoster virus (VZV) that entered the cutaneous nerves from a previous infection with varicella (chickenpox) usually in childhood [1].

Varicella infection usually results in lifetime immunity, and varicella reinfection is very rare; however, the incidence of herpes zoster increases significantly in individuals who have risk factors that suppress the immune system, such as old age, cancer especially hematological, human immunodeficiency virus (HIV), bone marrow or solid organ transplant recipients, taking immunosuppressive medications, female gender [2]. Moreover, people with chronic obstructive pulmonary disease (COPD), hypertension (HTN), diabetes mellitus, mental illnesses, osteoskeletal illnesses, and renal failure are more likely to develop herpes zoster [3].

Complications of herpes zoster can affect patient's quality of life and impair their social activities and sleep. The most common complication is post-herpetic neuralgia, which is a pain lasting for three to six months after the rash appears or persisting even after the rash has completely disappeared. Other complications include secondary bacterial infections, herpes zoster ophthalmicus, cerebral and peripheral nerve palsies, and segmental zoster paresis [3].

A study has confirmed that the seroprevalence of varicella in Saudi Arabia equals 86% [4]. Moreover, as the elderly population increases, there will be a corresponding increase in the number of people affected by herpes zoster [5].

The Advisory Committee on Immunization Practices (ACIP) recommended the recombinant zoster vaccine (RZV, Shingrix) to prevent shingles and its complications for individuals who are 50 years of age or older. In all age groups, effectiveness after two doses of the Shingrix vaccine was high. In a clinical trial with almost 30,000 participants, the vaccination was 96.6% effective in adults 50 to 59 years old, 97.4% effective in adults 60 to 69 years old, and 91.3% effective in people 70 years and above [6]. This means that the knowledge and acceptance of herpes zoster infection and its vaccination are crucial. Therefore, we are conducting this study to assess the current awareness and attitude toward HZ and its vaccination among individuals at risk in Saudi Arabia.

## Materials and methods

Study design and sample size

This cross-sectional study was conducted in Saudi Arabia between February 2023 and June 2023. We included all the Saudi people who had previous varicella zoster infection with one or more of herpes zoster risk factors: age above 50 years, people suffering from chronic disease (cancers, diabetes, inflammatory bowel disease, depression, rheumatoid arthritis, cardiovascular diseases, renal diseases, systemic lupus erythematous), patients who are on immunosuppressive medications, and we excluded those who didn't meet the inclusion criteria or refused to participate.

The estimated sample size required for the study was 331, which was calculated by Epi Info™ software version 2.1, considering the confidence interval of 95% and level of significance of 5%. We aimed to maximize the sample size to reach 2500 participants to increase the efficiency of the generalization of our results. However, we were able to collect the data from 1883 participants. The sample was distributed equally among the five regions of Saudi Arabia.

The study was carried out after approval from the Biomedical Research Ethics Committee of Umm Al-Qura University, Makkah City, Saudi Arabia, with approval no. HAPO-02-K-012-2023-03-1491. Informed consent was obtained from the participants before filling out the questionnaire.

Study tool

A structured, self-administered online questionnaire was used to collect data. The questionnaire was modified and adopted from another study [7]. An Arabic version was used to enhance the participants' understanding. The questionnaire was divided into four sections. The first section focused on demographic characteristics, like age, gender, educational level, area of residence, and participants' medical history. The second section included six questions to assess participants' general knowledge about herpes zoster. The third section included four questions to assess participants' knowledge about herpes zoster vaccination. The last section consisted of six questions to assess participants' attitudes towards the prevention of herpes zoster. The questionnaire consisted of true or false, multiple choice, and Likert scale questions.

Statistical analysis

The obtained data were statistically analyzed using SPSS version 22 (IMB Inc., Armonk, US). The mean, standard deviation, and significance utilizing the Chi-square test were used for measurements and comparative analysis. A significance level of <0.05 was considered statistically significant. Participants' overall knowledge was categorized based on the total percentage score as follows: 80% and above was considered as high, 60%-79% as intermediate, 40%-59% as moderate, 20%-39% as low, and a score less than 20% as unsatisfactory. A three-point Likert scale was used to assess the participants' attitudes towards HZ and its vaccine. 

## Results

A total of 1883 participants who met our inclusion criteria have completed the study survey. Most of the study participants, 1175 (62.4%), were females, while the rest, 708 (37.6%), were males. About more than half of them, 1038 (55.1%), were 20-30 years old, 374 (19.9%) were 31-40 years old, 286 (15.2%) were 41-50 years old, 116 (6.1%) were 51-60 years old and only 69 (3.7%) were more than 60 years old. Participants were almost equally distributed across the five regions of Saudi Arabia. The highest area of residence, 387 (20.6%), was in the central region, while the lowest area of residence, 286 (15.2%), was in the northern region. The majority of the participants, 1265 (67.2%), held a bachelor's degree, 402 (21.3%) had a high school degree, 140 (7.4%) had an education higher than a bachelor's degree, and 76 (4%) had below high school degree. Over half of the participants, 1062 (56%), had a positive history of chickenpox, 687 (36.5%) had a negative history of chickenpox, and 134 (7.1%) participants were not sure if they had a history of chickenpox. Most of the participants, 1486 (78.9%), heard about herpes zoster, and only 67 (3.6%) reported having a positive history of herpes zoster, while 1695 (90%) had a negative history of herpes zoster. Regarding herpes zoster risk factors among participants, 232 (12.3%) were at the age of 50 and above, 167 (8.9%) had diabetes mellitus, 114 (6.1%) had depression, 78 (4.1%) had cardiovascular disease, 74 (3.9%) had inflammatory bowel disease, 59 (3.1%) had rheumatoid arthritis, 44 (2.3%) were on immunosuppressive therapy, 23 (1.2%) had systemic lupus erythematosus and 21 (1.1%) had cancer (Table 1).

**Table 1:** Socio-demographic characteristics of study participants

Characteristic		Frequency	Percentage
Gender	Female	1175	62.4
	Male	708	37.6
Age	20 – 30 years	1038	55.1
	31 – 40 years	374	19.9
	41 – 50 years	286	15.2
	51 – 60 years	116	6.2
	More than 60	69	3.7
Area of residence	Central region	387	20.6
	Eastern region	339	18
	Western region	363	19.3
	Northern region	286	15.2
	Southern region	363	19.3
Educational level	Below high school	76	4
	High school	402	21.3
	Bachelor's degree	1265	67.2
	Higher education	140	7.4
History of chickenpox	Positive	1062	56
	Negative	687	36.5
	Unsure	134	7.1
Heard of herpes zoster	Yes	1486	78.9
	No	397	21.1
History of herpes zoster	Positive	67	3.6
	Negative	1695	90
	Unsure	121	6.4
Assessment of herpes zoster risk factors	Age of 50 or above	232	12.3
	Cardiovascular disease	78	4.1
	Diabetes mellitus	167	8.9
	Systemic lupus erythematosus	23	1.2
	Rheumatoid arthritis	59	3.1
	Kidney disease	36	1.9
	Inflammatory bowel disease	74	3.9
	Cancer	21	1.1
	Depression	114	6.1
	Immunosuppressive therapy	44	2.3

Table 2 shows the participants' responses to the questions regarding the knowledge of herpes zoster. To the question if an individual had chickenpox, would he/she be at risk of HZ, 483 (25.7%) agreed, and 172 (9.1%) disagreed, while 1228 (65.2%) didn't know the answer. With the statement that immunocompromised individuals are at a higher risk of HZ, 925 (49.1%) agreed, 74 (3.9%) disagreed, and 884 (46.9%) didn't know the answer. With the statement that young people will not have HZ, 210 (11.2%) agreed, 604 (32.1%) disagreed, and 1069 (56.8%) didn't know the answer. When we asked that the individuals who have contact with HZ patients will acquire HZ, 547 (29%) agreed with it, 404 (21.5%) disagreed, and 932 (49.5%) didn't know the answer. With the statement that there are no drugs available for treating HZ, 136 (7.2%) agreed, 864 (45.9%) disagreed, and 883 (46.9%) didn't know the answer. When we asked whether the respondents knew any symptoms of HZ, they replied rash, 1587 (84.3%), as well as neuropathic pain, 935 (49.7%), blindness, 313 (16.6%), hearing loss, 249 (13.2%), death, 374 (19.9%).

**Table 2:** Responses to questions regarding the knowledge of herpes zoster HZ - herpes zoster

Item	Answered "true"		Answered "false"		Answered "I don’t know"	
	n	%	n	%	n	%
If an individual has had chickenpox, he/she will be at risk of HZ	483	25.7	172	9.1	1228	65.2
Immunocompromised individuals are at a higher risk of HZ	925	49.1	74	3.9	884	46.9
Young people will not have HZ	210	11.2	604	32.1	1069	56.8
Individuals who have contact with HZ patients will acquire HZ	547	29	404	21.5	932	49.5
There are no drugs available for treating HZ	136	7.2	864	45.9	883	46.9
Do you know any symptoms of HZ (more than one can be selected)						
Rash	1587	84.3	296	15.7	-	-
Neuropathic pain	935	49.7	948	50.3	-	-
Blindness	313	16.6	1570	83.4	-	-
Hearing loss	249	13.2	1634	86.8	-	-
Death	374	19.9	1509	80.1	-	-

Table 3 shows the responses to questions regarding the knowledge of herpes zoster vaccination. More than half of the participants, 1112 (59.1%), believed that the HZ vaccine can reduce the incidence of disease by >50%, and 672 (35.7%) believed that the HZ vaccine can treat active HZ. To the question about the age group (in years) that is approved for vaccination against HZ, 284 (15.1%) answered 18 or more, 493 (26.2%) answered 50 or more, 385 (20.4%) answered that there is no age limit, 721 (38.3%) didn't know the answer. When we asked by which group(s) of people HZ vaccination can be taken, 308 (16.4%) thought that by those who did not have/or were unsure about a history of chickenpox, 422 (22.4%) thought that those who had chickenpox but no HZ, 126 (6.7%) had HZ before, and 1027 (54.5%) didn't know the answer to this question.

**Table 3:** Responses to questions regarding the knowledge of herpes zoster vaccination HZ - herpes zoster

Items	Answered "true"		Answered "false"		Answered "I don’t know"	
	n	%	n	%	n	%
HZ vaccine can reduce the incidence of disease by >50%	1112	59.1	47	2.5	724	38.4
HZ vaccine can treat active HZ	672	35.7	245	13	966	51.3
Which age group (in years) is approved for vaccination against HZ?						
18 or more	284	15.1	-	-	-	-
50 or more	493	26.2	-	-	-	-
There is no age limit	385	20.4	-	-	-	-
I do not know	721	38.3	-	-	-	-
HZ vaccination can be taken by which group(s) of people (more than one can be selected except the last choice)						
Did not have/unsure of the history of chickenpox	308	16.4	-	-	-	-
Had chickenpox, but no HZ	422	22.4	-	-	-	-
Had HZ before	126	6.7	-	-	-	-
I do not know	1027	54.5	-	-	-	-

**Figure 1:**
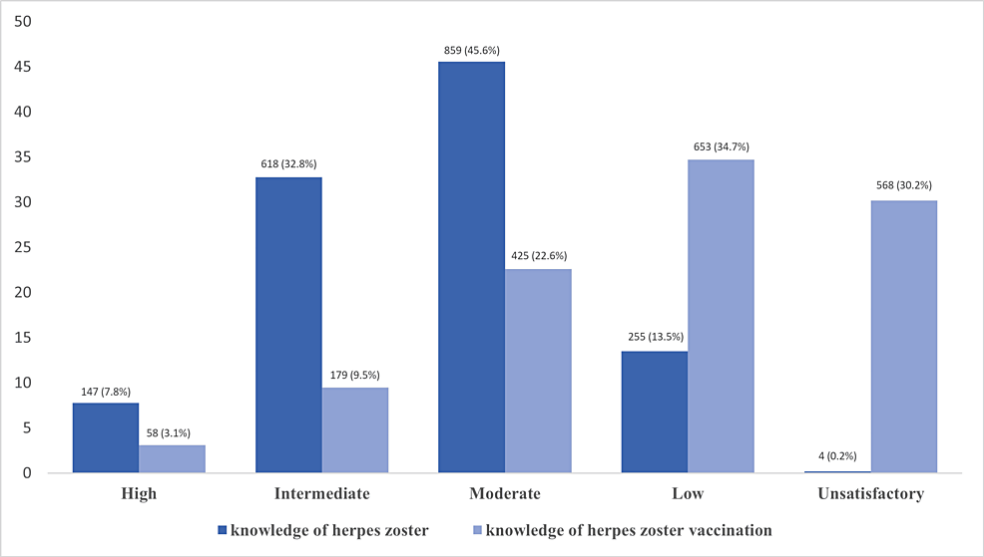
Knowledge of herpes zoster and its vaccination

Figure 1 shows the total results of the assessment of the knowledge about HZ and its vaccination. It shows that only 7.8% and 3.1% of the respondents had high knowledge regarding HZ and its vaccination, respectively.

Table 4 shows participants' responses to questions regarding their attitude toward the prevention of herpes zoster. Most of the participants, 852 (45.2%), disagreed that they have an adequate understanding of HZ, more than half of the participants, 1203 (63.9%), agreed that HZ has a significant effect on health, 675 (35.8%) worried that they have HZ, 1358 (72.1%) answered that they have an interest in knowing more about HZ, 519 (27.6%) respond that they have adequate channels in knowing how to prevent HZ, and 1352 (71.8%) are interested in knowing more about the prevention of HZ.

**Table 4:** Responses to questions regarding the attitude toward the prevention of herpes zoster HZ - herpes zoster

Items	Answered "agree"		Answered "disagree"		Answered "neutral"	
	n	%	n	%	n	%
I have an adequate understanding of HZ	367	19.5	852	45.2	664	35.3
HZ has a significant effect on health	1203	63.9	222	11.8	458	24.3
I am worried about having HZ	675	35.8	639	33.9	569	30.2
I am interested in knowing more about HZ	1358	72.1	171	9.1	354	18.8
I have adequate channels in knowing how to prevent HZ	519	27.6	689	36.6	675	35.8
I am interested in knowing more about the prevention of HZ	1352	71.8	168	8.9	363	19.3

Participants who showed a high level of knowledge, according to gender, constituted 97 females (66%) and 50 males (34%). According to age, 96 (65.3%) were 20-30 years, 20 (13.6%) were 31-40 years, 17 (11.6%) were 41-50 years, seven (4.8%) were 51-60 years, and seven (4.8%) were older than 60. According to the region of residence, most of the participants with a high level of knowledge were from the western region, 56 (38.1%), followed by the central region, 30 (20.4%). According to the educational level, the respondents with bachelor's degree had a high level of knowledge, 95 (64.6%), followed by participants with high school degree, 30 (20.%). According to the risk for herpes zoster, 98 (66.7%) were at high risk, while 49 (33.3%) were at low risk. Of participants who showed an intermediate level of knowledge, 409 (66.2%) were female, and 209 (33.8%) were male. According to the age, 343 (55.5%) were 20-30 years, 117 (18.9%) were 31-40 years, 108 (17.5%) were 41-50 years, 27 (4.4%) were 51-60 years, and 23 (3.7%) were older than 60. According to the region of residence, most of the participants with an intermediate level of knowledge were from the western region, 176 (28.5%), followed by the central region, 136 (22%). According to the educational level, the majority had a bachelor's degree, 421 (68.1%), 125 (20.2%) had a high school degree, 52 (8.4%) had a higher education degree, and 20 (3.2%) had a degree below high school. According to the risk for herpes zoster, 429 (69.4%) were at high risk, while 189 (30.6%) were at low risk. Of participants who showed a moderate level of knowledge, 521 (60.7%) were female, and 338 (39.3%) were male. According to the age, 454 (52.9%) were 20-30 years, 174 (20.3%) were 31-40 years, 130 (15.1%) were 41-50 years, 68 (7.9%) were 51-60 years, and 33 (3.8%) were older than 60. According to the region of residence, most of the participants with a moderate level of knowledge were from the western region 221 (26.7%), followed by the central region 188 (21.9%). According to the educational level, the majority, 591 (68.8%), had a bachelor's degree, 171 (19.9%) had a high school degree, 61 (7.1%) had a higher education, and 36 (4.2%) had a degree below high school. According to the risk for herpes zoster, 624 (72.6%) were at high risk, while 235 (27.4%) were at low risk. Of the participants who showed a low level of knowledge, 146 (57.3%) were female, and 109 (42.7%) were male. According to age, 142 (55.7%) were 20-30 years, 62 (24.7%) were 31-40 years, 30 (11.8% %) were 41-50 years, 14 (5.5%) were 51-60 years, and six (2.4%) were older than 60. According to the region of residence, the majority of participants with a low level of knowledge were from the eastern region 62 (24.3%), followed by the northern region 58 (22.7%). According to the educational level, 155 (60.8%) had a bachelor's degree, 75 (29.4%) had a high school degree, 14 (5.5%) had a higher education degree, and 11 (4.3%) had a degree below a high school. According to the risk for herpes zoster, 143 (56.1%) were at high risk, while 112 (43.9%) were at low risk (Table 5).

**Table 5:** Knowledge of HZ in association with demographic characteristics of the participants * Crosstabs were used; the difference is considered significant at a p-value ≤0.05. HZ - herpes zoster

Demographic characteristics	Knowledge of herpes zoster				p-value
	High	Intermediate	Moderate	Low/unsatisfactory	
Gender					
Male	50 (34%)	209 (33.8%)	338 (39.3%)	111 (42.9%)	0.033*
Female	97 (66%)	409 (66.2%)	521 (60.7%)	148 (57.1%)	
Age group					
20 – 30 years	96 (65.3%)	343 (55.5%)	454 (52.9%)	145 (56%)	0.016*
31 – 40 years	20 (13.6%)	117 (18.9%)	174 (20.3%)	63 (24.3%)	
41 – 50 years	17 (11.6%)	108 (17.5%)	130 (15.1%)	31 (12%)	
51 – 60 years	7 (4.8%)	27 (4.4%)	68 (7.9%)	14 (5.4%)	
More than 60	7 (4.8%)	23 (3.7%)	33 (3.8%)	6 (2.3%)	
Region of residence					
Central region	30 (20.4%)	136 (22%)	188 (21.9%)	33 (12.7%)	<0.001*
Eastern region	26 (17.7%)	107 (17.3%)	144 (16.8%)	62 (23.9%)	
Western region	56 (38.1%)	176 (28.5%)	221 (25.7%)	55 (21.2%)	
Northern region	7 (4.8%)	82 (13.3%)	137 (15.9%)	60 (23.2%)	
Southern region	28 (19%)	117 (18.9%)	169 (19.7%)	49 (18.9%)	
Educational level					
Below high school	9 (6.1%)	20 (3.2%)	36 (4.2%)	11 (4.2%)	0.058
High school	30 (20.4%)	125 (20.2%)	171 (19.9%)	76 (29.3%)	
Bachelor's degree	95 (64.6%)	421 (68.1%)	591 (68.8%)	158 (61%)	
Higher education	13 (8.8%)	52 (8.4%)	61 (7.1%)	14 (5.4%)	
Risk for herpes zoster					
High risk	98 (66.7%)	430 (69.4%)	624 (72.6%)	145 (56%)	<0.001*
Low risk	49 (33.3%)	188 (30.6%)	235 (27.4%)	114 (44%)	

## Discussion

The Centers for Disease Control and Prevention (CDC) recommends that adults aged 50 years and older, besides immunocompromised adults aged 19 years or older, should receive two doses of shingles vaccine (Shingrix) to prevent shingles and its complications [8]. The protection of the general population necessitates the application of global disease control and prevention recommendations, which can't be fulfilled unless the population gains adequate knowledge about the disease sequelae. Nevertheless, the knowledge level of herpes zoster (shingles) hasn't yet been investigated thoroughly among the entire country of Saudi Arabia, including those who are at higher risk of the disease.

Therefore, we intended to assess the knowledge and attitude towards HZ and its vaccination in this study. The majority of our participants exhibited a moderate (45.6%) to intermediate (32.8%) understanding of the disease, which is better than the United Arab Emirates (UAE) study, where only 60% were aware of HZ [9]. However, most participants were unaware of the HZ vaccine, which is similar to the UAE study findings [9]. Interestingly, more than half of the respondents either had a history of chickenpox or had heard about the disease, while mostly half of them carry one or more of the shingles risk factors, which is contrary to the level of awareness of the vaccine. Thus, comprehensive details about HZ and its complications should be elaborated by the treating physician, including the means of prevention and vaccination. Vaccines have been able over the years to reduce mortality and morbidity of numerous diseases [10].

Our findings contrast with another study done in South Korea, showing that 80% and more than half of their sample were adequately aware of HZ and its vaccine, respectively [11]. Campaigns and public awareness programs should be activated and enhanced to improve the population's awareness of HZ, rectify the negative attitudes towards the HZ vaccine, and reinforce willingness to receive the vaccine. A United States study found a positive relationship between the adequate understanding of HZ and its vaccine and the individual's willingness to get the vaccine [11].

Subjects who were knowledgeable about the disease and its vaccine were mostly of higher education, which is in accordance with Hong Kong and UAE studies [7,9]. Hence, the population should be encouraged and provided with sufficient support for gaining a higher education as it's associated with better knowledge about HZ, which in turn helps prevent the disease and its complications.

Most of the respondents acknowledged the effect of HZ on public health and showed interest in learning more about the disease and its prevention, regardless of their current level of awareness. However, around half of the participants revealed that they don't have enough channels to get more information about HZ prevention. The most available sources reported by the UAE study are family and friends or the internet, which are not completely reliable [9]. Henceforth, healthcare centers and medical professionals should offer more reliable sources to provide evidence-based information about HZ and its prevention.

Strengths and limitations

Our study provides conclusive evidence of the current level of awareness about HZ and its vaccine, which has not been discussed thoroughly in Saudi Arabia before. However, we used a convenience sampling technique, which affects the quality of the randomization of our study sample. Additionally, recall bias is possible as respondents were asked to self-report their history of chickenpox, HZ, and a number of chronic diseases. Despite that, the results of our study are valid and can be generalized to the total number of the targeted population among all the regions of Saudi Arabia, as our study sample was almost equally distributed among the regions of the country.

## Conclusions

Herpes zoster is a viral infection resulting from the reactivation of the varicella-zoster virus. Recently, the Saudi Ministry of Health recommended the herpes zoster vaccination and provided it in primary health centers for individuals over the age of 50. The general population of Saudi Arabia had inadequate knowledge about herpes zoster and its vaccine. We observed a positive attitude toward the herpes zoster vaccine, and reporndents knew how effective the vaccine was. Subsequently, awareness and education programs targeting individuals at high risk of herpes zoster are required to enhance awareness and knowledge about herpes zoster and to improve their attitudes toward the herpes zoster vaccination. By doing this, we can hopefully increase the administration of the herpes zoster vaccine and reduce the number of herpes zoster infections and its complications.
